# Conformation and Dynamics of the Cyclic Lipopeptide Viscosinamide at the Water-Lipid Interface

**DOI:** 10.3390/molecules24122257

**Published:** 2019-06-17

**Authors:** Niels Geudens, Benjámin Kovács, Davy Sinnaeve, Feyisara Eyiwumi Oni, Monica Höfte, José C. Martins

**Affiliations:** 1 NMR and Structural Analysis Unit, Department of Organic and Macromolecular Chemistry, Ghent University, Campus Sterre, S4, Krijgslaan 281, B-9000 Gent, Belgium; niels.geudens@ugent.be (N.G.); benjamin.kovacs@ugent.be (B.K.); davy.sinnaeve@ugent.be (D.S.); 2 CNRS, UMR 8576 Unité de Glycobiologie Structurale et Fonctionnelle, Université de Lille, 59000 Lille, France; 3 Laboratory of Phytopathology, Department of Plants and Crops, Ghent University, Coupure Links 653, B-9000 Gent, Belgium; FeyisaraEyiwumi.Olorunleke@UGent.be (F.E.O.); monica.hofte@ugent.be (M.H.)

**Keywords:** pseudomonas, viscosinamide, conformation, NMR spectroscopy, isotopic labelling

## Abstract

Cyclic lipodepsipeptides or CLiPs from *Pseudomonas* are secondary metabolites that mediate a wide range of biological functions for their producers, and display antimicrobial and anticancer activities. Direct interaction of CLiPs with the cellular membranes is presumed to be essential in causing these. To understand the processes involved at the molecular level, knowledge of the conformation and dynamics of CLiPs at the water-lipid interface is required to guide the interpretation of biophysical investigations in model membrane systems. We used NMR and molecular dynamics to study the conformation, location and orientation of the *Pseudomonas* CLiP viscosinamide in a water/dodecylphosphocholine solution. In the process, we demonstrate the strong added value of combining uniform, isotope-enriched viscosinamide and protein NMR methods. In particular, the use of techniques to determine backbone dihedral angles and detect and identify long-lived hydrogen bonds, establishes that the solution conformation previously determined in acetonitrile is maintained in water/dodecylphosphocholine solution. Paramagnetic relaxation enhancements pinpoint viscosinamide near the water-lipid interface, with its orientation dictated by the amphipathic distribution of hydrophobic and hydrophilic residues. Finally, the experimental observations are supported by molecular dynamics simulations. Thus a firm structural basis is now available for interpreting biophysical and bioactivity data relating to this class of compounds.

## 1. Introduction

Many *Pseudomonas* and *Bacillus* spp. produce cyclic lipodepsipeptides (CLiPs) via multi-domain, non-ribosomal peptide synthetases [1]. *Pseudomonas* CLiPs are involved in several secondary functions, such as cell motility, adhesion and biofilm formation [2,3,4], or ecological functions, such as promoting plant-growth [2,5] or triggering a defense response in plants [4,6]. They have also been reported to display a range of antagonistic properties, such as antifungal [7], antibiotic [8,9], insecticidal [10], antiviral [11] and anti-oomycete activity. [12] Antitumor activity has also been reported [13,14,15]. Recently, the antimicrobial activities of *Pseudomonas* CLiPs (Ps-CLiPs) were thoroughly reviewed [16]. 

**Figure 1:**
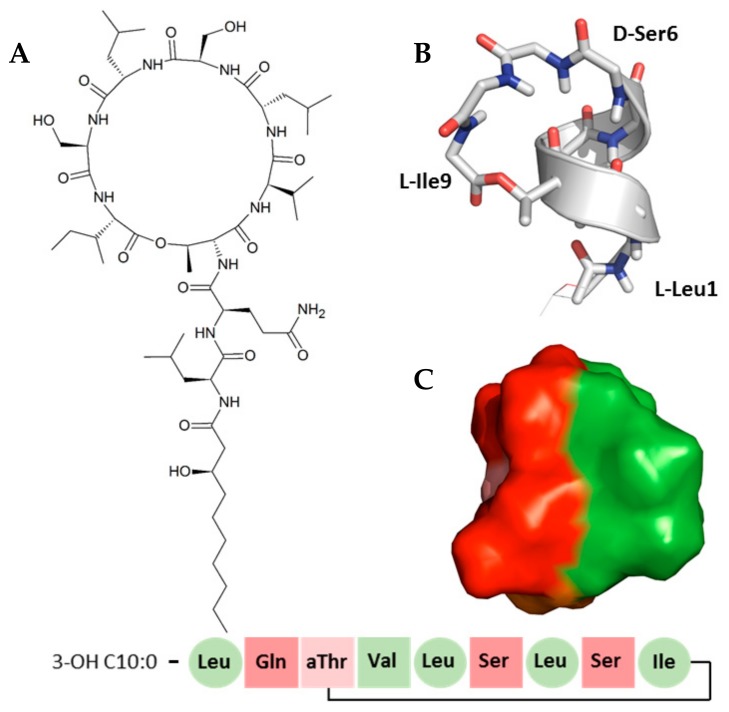
(**A**) Chemical structure of viscosinamide A. (**B**) Solution conformation of viscosinamide A showing a left-handed helix from l-Leu1 to d-Ser^6^ followed by a loop. (**C**) Arrangement of hydrophobic (green) and hydrophilic (red) side chains in a solvent accessible (Connolly) surface representation of the solution structure of viscosinamide A. In both B and C, the flexible fatty acid side-chain beyond the C4 position has been omitted for clarity. The amino acid sequence is detailed at the bottom, whereby the amino acids colors indicate the polarity of the amino acids (red = polar, green = hydrophobic), and the shape indicates the stereochemistry (sphere = l-amino acid, square = d-amino acid).

The chemical blueprint of Ps-CLiPs is illustrated with that of viscosinamide A in Figure 1A. They are composed of an oligopeptide, cyclized through a lactone (depsi) bond, and capped at the N-terminus by a fatty acid moiety [1,17,18]. Up till now, well over 100 CLiPs originating from *Pseudomonas* spp. have been described with varying levels of structural and biological activity details [16]. This wide variety of *Pseudomonas* CLiPs can be categorized into distinct groups based on oligopeptide length, amino acid sequence and macrocycle size, each group being named after a particular prototype sequence. With more than 20 members, the viscosin group represents the largest and most extensively characterized CLiP-group. Viscosins are nonapeptides cyclized via an ester bond between the C-terminal carbonyl and the side chain of a d-*allo*-Thr (d-aThr) at position 3, thus leading to a 7-residue macrocycle (Figure 1). 

Their N-terminal fatty acid cap is, in most cases, a (*R*)-3-hydroxydecanoic acid moiety [19]. Notable members of the viscosin group include viscosin itself, the viscosinamides, the pseudodesmins, WLIP, the massetolides and the pseudophomins [16]. The viscosin group sequences show the same pattern of hydrophobic and hydrophilic residues, the most significant differences amongst viscosin-group CLiPs being either the presence of a d-Glu or d-Gln at position 2, and/or a d- or l-Leu at position 5, generating four viscosin-subgroups [19]. Importantly, sequences containing a d-Gln (such as viscosinamide and pseudodesmin) are neutral compounds, whereas viscosin and WLIP have a d-Glu and carry a single negative charge. In the majority of cases, the uncharged Q-subgroup CLiPs are more active against Gram-positive bacteria than the charged E-subgroup ones. In contrast, a clear effect was found in the antimicrobial activity that reflects the d/l stereo-inversion of the Leu5 residue [19]. Previous investigations have proposed that interaction with the cellular membrane is most likely at the basis of mode of action of CLiPs. Using a total synthesis approach, we have provided support for this view by showing that the mirror image of pseudodesmin A is equally active as the natural compound when tested against a panel of Gram-positive bacteria, thus eliminating a chiral receptor-based interaction as the cause of its antimicrobial bioactivity [20]. In addition, fluorescence permeability assays on model membranes demonstrated that the four subgroup viscosin CLiPs mentioned above induce permeabilization, the neutral CLiPs being somewhat more potent (C_1/2_ ~3.7 µM) than the charged ones (C_1/2_ ~4.9 µM). These observations complemented earlier reports on individual viscosin CLiPs in this respect [21,22]. 

The solution structures determined previously by NMR for pseudodesmin A and viscosinamide A in acetonitrile [23,24], combined with the crystal structure of WLIP [25], pseudodesmin A [9] and pseudophomins [26] have revealed that all of these viscosin group members share the same overall fold: a left handed α-helix between l-Leu1 and d-Ser6 proceeding into a loop that connects the C-terminus to the middle of the α-helix via an ester (or depsi) bond with the side chain of d-aThr3 (Figure 1B). This particular fold creates a clear separation between hydrophilic and hydrophobic residues (Figure 1C) leading to an amphipathic surface, thus equipping the molecule with suitable physicochemical properties to interact with a membrane surface. To better understand the molecular aspects of the peptide-membrane interaction and use these to improve our understanding of the mode of action, knowledge of the conformation adopted by the CLiPs at the water-lipid interface is indispensable.

Here, we report on our investigation of this interaction using viscosinamide A (VA) as CLiP of interest and the water solution of zwitterionic dodecylphosphocholine (DPC), a micellar membrane mimicking detergent. Micelles can be considered the simplest model membrane system. However, detergents typically create a more dynamic environment than the native bilayer. In this way, they may impart flexibility and instability to the interacting membrane peptides. Micelles also possess a high surface curvature, typically not found in bilayers. Nevertheless, DPC micelles provide a reasonable model of the lipid environment of a cell membrane and are therefore well-established for use in NMR investigations at a water-lipid interface [27,28]. We first establish the partitioning of VA across the water and lipid phase using diffusion NMR spectroscopy. Then, in spite of strong limitations to obtain a nOe-based ^1^H–^1^H distance restraint data set for structure determination in the DPC solution, we demonstrate that the conformation determined in acetonitrile solution is fully maintained. Particularly novel in our approach is the use of ^15^N or ^13^C/^15^N isotope-enriched VA which allows the application of dedicated NMR techniques typically reserved for recombinantly expressed proteins [29]. By using the long-range HNCO experiments [30] we were able—we believe for the first time—to directly identify the donor and acceptor moieties involved in hydrogen bond formation in a non-ribosomal peptide metabolite. Finally, by using both water soluble and lipid anchored paramagnetic labels we established the location and orientation of VA molecules with respect to the DPC-water interface. The results were confronted with MD simulations of VA in explicit DPC + H_2_O mixture solution so as to obtain an emerging picture of the behavior of neutral viscosin-group CLiPs at the water-membrane interface. This is the first such combined study for any *Pseudomonas* CLiP and thus provides the first structural insight for this large group of compounds into the interaction with its primary target.

## 2. Results

### 2.1. Initial Observations and Partitioning of VA in DPC Solution Using Diffusion NMR Spectroscopy

To investigate the behavior of VA in the presence of DPC micelles, a 100 mM DPC solution in phosphate buffer at pH 7.4 was used throughout. Using the aggregation number N_aggr_ of 56 ± 6 [31], the total DPC micelle concentration was estimated to be ~1.8 mM prior to VA addition. Since VA is insoluble in water—at least at levels allowing it to be monitored by NMR spectroscopy—the DPC solution was added to dry amounts of VA in order to cover peptide to lipid ratio’s (P:L) from 1:100 to 7:100. Assuming the micelles and N_aggr_ are not significantly perturbed by the addition of VA, this corresponds to a variation in peptide to micelle ratio (P:M) of 0.6:1 to 3.9:1. A single set of resonances was observed in all cases, with identical chemical shifts that are insensitive to P:L (Supplementary Figure S1). Compared to the NMR spectra in acetonitrile [23], uniform broadening of all VA resonances is clearly apparent (Supplementary Figure S2). The line broadening and chemical shifts remained constant over the titrated P:L range. Irrespective of the particular chemical exchange kinetics (either fast or slow) on the NMR frequency time scale, the observation of a single set of VA resonances with chemical shifts independent of the P:L clearly indicates that partitioning of VA in the DPC is already highly pronounced towards the lipid phase at the lowest P:L. This was confirmed using diffusion NMR spectroscopy, a well-established technique to investigate partitioning of molecules in a water/lipid dispersion through the diffusion coefficient of the solute molecules [32,33,34,35]. In the case at hand, difficulties in extracting the DPC diffusion coefficient from overlapping VA and DPC resonances were avoided by taking advantage of the fact that only DPC contributes to the ^31^P-NMR spectrum. Thus, ^31^P diffusion NMR spectroscopy was used to selectively measure the DPC diffusion coefficient while that of VA was monitored from non-overlapping amide resonances in the ^1^H-NMR spectrum. In the absence of VA, the DPC micelles display a diffusion coefficient of 90.1 ± 0.7 µm^2^/s. Upon addition of VA, the diffusion coefficient of DPC molecules decreases to 79.6 ± 4.0 µm^2^/s while that of VA is found to be 80.9 ± 0.5 µm^2^/s, showing the values are identical within error in the solution. We conclude that the incorporation of VA into the DPC lipid environment is close to 100%, creating VA + DPC aggregates with a hydrodynamic radius that is on average ~12% larger compared to that of intact DPC micelles at the same detergent concentration. The line broadening of the VA ^1^H resonances are therefore to be interpreted as resulting from increased T_2_ relaxation caused by the reduced rotational tumbling upon its association with the DPC micelles, the hydrodynamic diameter of which can be estimated to be 3.1 nm. 

### 2.2. The Conformation of VA Observed in Acetonitrile is Preserved in DPC Solution

Initially, 2D NOESY was used to qualitatively assess the impact of the interaction with DPC micelles on the conformation of VA. As a consequence of the hindered rotational dynamics of the peptide, strong negative nOe cross-peaks could be observed, in contrary to the weaker, positive nOes measured in acetonitrile [23]. Comparison of the nOe correlations involving backbone H^N^ and H^α^ nuclei in both conditions shows that the main characteristics of the fold are preserved: distinct nOe contacts typical for the formation of the left-handed α-helix remain present for VA in the DPC solution (data not shown). Based on these, we infer that the conformation of VA appears maintained when the lipopeptide is inserted in the micellar DPC environment. No intermolecular nOe contacts were observed between VA and the residual ^1^H resonances of the DPC molecules, which is not unexpected given the high degree of deuteration of the detergent. 

Unfortunately, the NOESY data proved intractable to generate reliable ^1^H–^1^H distance constraints for a three-dimensional structure determination as considerable spin-diffusion was apparent, even at shorter mixing times. Resorting to 2D ROESY instead—which is less sensitive to spin-diffusion—proved equally unsuccessful, since no significant rOe cross-peak build-up could be achieved due to efficient T_1ρ_ relaxation during the spin lock period, even when the mixing time was trimmed down to 20 ms. Three-bond scalar coupling constants (^3^*J*) constitute another set of parameters related to conformation that can be accessed by NMR spectroscopy [36,37,38]. In particular, the phi-angle of each residue is accessible through the ^3^*J*_HNHα_ coupling constant, thus providing information on the backbone conformation. Therefore, local changes in this conformation can be judged from the confrontation of the respective ^3^*J*_HNHα_ values for each residue measured in acetonitrile and in DPC solution. In acetonitrile, the ^3^*J*_HNHα_ coupling constants (collected in Table 1) can be read directly from the splitting of the amide H^N^ resonances. In DPC solution however, the aforementioned line-broadening precludes such access. All in all, only limited information concerning the conformation of VA in a DPC micellar solution appeared to be available from ^1^H-NMR spectroscopy, undermining our goal to derive the conformation of VA in a water/lipid environment through restraint-based structure calculations. 

This setback was successfully countered by use of ^15^N and ^13^C/^15^N isotope-enriched VA in combination with various NMR methodologies well-known for the study of protein structures in solution [39,40]. To achieve the isotope enrichment, the VA producing *Pseudomonas* sp. DR54 strain was grown on M9 minimal medium supplemented with uniformly ^13^C-enriched d-glucose and/or ^15^N-enriched ammonium chloride as sole carbon and nitrogen source, respectively. Growth was found to be effective under these conditions, as also reported previously for viscosin [13]. Successful incorporation of ^15^N at levels above 95% was easily demonstrated from the integral of ^15^N satellites relative to the central resonance in the 1D ^1^H-NMR spectra (Supplementary Figure S3). A similar approach could not be applied to the degree of ^13^C incorporation due to signal overlap. However, it could be established from mass spectrometric data instead (see Materials and Methods). Isotope enrichment itself does not remedy the line broadening and spin diffusion issues mentioned above; however, the ^15^N-enrichment of the peptide allows the fast, quantitative measurement of the ^3^*J*_HNHα_ coupling constants via the HNHA experiment (see Materials and Methods) [41]. The ^3^*J*_HNHA_ values extracted for VA in both acetonitrile and DPC solution using the HNHA experiment are collected in Table 1. Comparison of the ^3^*J*_HNHA_ values in acetonitrile solution obtained from the splitting of the H^N^ signals in the 1D ^1^H spectrum with those derived from the HNHA shows excellent agreement: the difference between the two data sets yields a RMSD of 0.09 Hz. This clearly shows equivalence of both methods and discards any concerns for method bias in comparing acetonitrile data with that in DPC solution, where only the HNHA could be used.

The difference in ^3^*J*_HNHA_ values for VA in DPC solution with those in acetonitrile, both extracted from the respective HNHA spectra, yields a RMSD of 0.28 Hz, indicating very good agreement. The largest absolute deviation—~0.5 Hz—remains modest in size and is observed for l-Leu1 and d-Gln2. This may reflect a small conformational change induced by insertion of the N-linked acyl chain in the DPC lipid phase. To explore the impact of the measured deviations, the φ angles were calculated from the experimental ^3^*J*_HNHA_ to obtain the difference Δφ associated with the difference in *J* observed between both environments. Since multiple φ values may satisfy a single ^3^*J*_HNHA_ we used the solution structure of VA in acetonitrile to select the closest calculated φ value. From Table 1, one can see that Δφ is very moderate, being less than 7° in all cases with the average absolute difference over all residues amounting to 2.3° only. Thus, based on the comparison of the ^3^*J*_HNHA_ values and the φ angles derived from them, we conclude that the backbone conformation as determined in acetonitrile is preserved in DPC solution and does not experience significant structural change. This is further substantiated by MD simulations performed for VA in either explicit acetonitrile or in a DPC + H_2_O environment (vide infra). Mutual comparison of the average dihedral phi angles for each residue in both environments over the entire 400 ns trajectory shows these to be within a few degrees of each other, the most notable exception again being observed for l-Leu1 (Δφ 26.6°) and to a lesser extent l-Ile9 (Δφ 15.9°) (Supplementary Table S1). While the difference in average angle Δφ for l-Leu1 is larger than expected from the difference in experimentally observed ^3^*J*_HNHA_ values, the MD results nevertheless support quasi-identical conformations in both environments with some localized change at the *N*-acyl-l-Leu1 position. This is in line with previous CD and FT-IR measurements on VA in POPC vesicles, where it was established that the conformation of VA is not altered upon membrane insertion [19].

### 2.3. Explicit Detection of Hydrogen Bonds Demonstrates the Rigidity of the Viscosinamide Fold

The availability of doubly ^13^C-,^15^N-enriched VA provides a unique opportunity to establish the presence and identity of hydrogen bonds in a direct way, instead of inferring these from geometric arguments using the structure itself. Such direct detection of hydrogen bonds between donor and acceptor groups is well-established for small to mid-sized isotope enriched proteins and nucleic acids but has to the best of our knowledge not been used for non-ribosomally produced peptides such as VA [43]. It exploits the fact that scalar couplings are active between the ^13^C and ^15^N nuclei on both sides of the hydrogen bond to develop a correlation that can be measured using 2D or 3D triple resonance NMR methods. In case of proteins, these small ‘through H-bond’ scalar couplings (^3h^*J*_NC′_) are used in a long-range HNCO type experiment, to connect the frequencies of the donor amide (*i*) ^15^N–^1^H nuclei to the carbonyl ^13^C′ of the accepting amide (*j*) C=O functional group [43,44]. In addition, the quantification of the ^3h^*J*_NiC′j_ values also affords a qualitative estimation of the hydrogen bond lifetime. Indeed, to generate a measurable cross-peak intensity, the hydrogen bond should be persistent at least for a time equal to ~1/^3h^*J*_NiC′j_. Given their small absolute size (<$\left|1\right|$ Hz), this requires a persistence on the order of tens of milliseconds to seconds. As a result, the detection of hydrogen bonds—either individually or as part of a larger network —allows to demonstrate the persistence of the local conformation in the overall structure of the molecule. 

**Figure 2:**
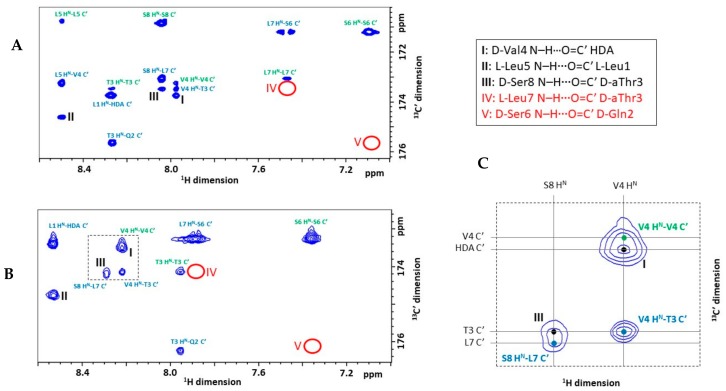
(**A**) Selected region of the long-range HNCO spectrum of VA in acetonitrile (700 MHz ^1^H frequency, 298.0 K) and (**B**) in DPC solution (700 MHz ^1^H frequency, 310.0 K). Intraresidual cross-peaks resulting from insufficiently suppressed ^1^*J*_NC′_ correlations are labeled in blue, cross-peaks between neighboring residues resulting from ^2^*J*_NC′_ correlations are labeled in green. Cross-peaks signifying hydrogen bond interactions are marked with roman numbers in black. The positions where the cross-peak of an assumed hydrogen bond is absent are highlighted with red roman numbers and red circles. (**C**) In DPC solution the cross-peaks of hydrogen bonds I and III overlap with the usual through-bond ^2^*J*_NC′_ and ^1^*J*_NC′_ couplings, respectively. For better visibility, the dashed area indicated in Figure 2B is enlarged here.

The small value of the ^3h^*J* couplings also limits the detection of hydrogen bonds from small to midsize proteins as the more efficient T_2_ relaxation of larger structures dissipates the contribution of the associated cross-peaks. To optimize the experimental parameters, we first recorded a long-range HNCO on VA in acetonitrile, where such relaxation-induced loss is of no concern. Owing to the much simpler complexity of VA compared to proteins, all desired information could already be extracted from a single, well-resolved 2D H^N^–C′ plane. Recording of the usual 3^rd 15^N frequency dimension could be omitted, strongly reducing the experimental time required (see Materials and Methods). The spectrum displays multiple cross-peaks that connect an amide ^1^H to a C′ resonance of the preceding or the same residue. These result from the usual through-bond ^1^*J*_Ni+1C′i_ or ^2^*J*_NiC′i_ scalar couplings, respectively, where the former are incompletely suppressed while the latter cannot be removed because they are of similar size to the desired ^3h^*J*_NiC′j_ ones. However, three cross-peaks stand out in the spectrum and allow to confirm the presence of three persistent backbone hydrogen bonds in acetonitrile solution: d-Val4 *N*–H···O=C′ HDA (I), l-Leu5 N–H···O=C′ l-Leu1 (II) and d-Ser8 N–H···O=C′ D-aThr3 (III) (Figure 2A). The former two represent the NH_i+4_ to CO_i_ hydrogen bonds typically present in α-helices. They independently support the presence of the helical moiety and clearly establishes the involvement of the exocyclic peptide chain in it, as already proposed from the previously reported NMR solution structure (Figure 1B). The NH_i+5_ to CO_i_ contact III is consistent with the fact that from d-Ser6/l-Leu7 onwards, the left-handed α-helical conformation is disrupted as the backbone folds back towards the side-chain of d-aThr3 as dictated by the depsi bond. The same change in backbone conformation prevents an l-Leu7 N–H to O=C′ d-aThr3 hydrogen bond from forming, supported by the absence of a corresponding cross-peak in the long-range HNCO spectrum.

**Figure 3:**
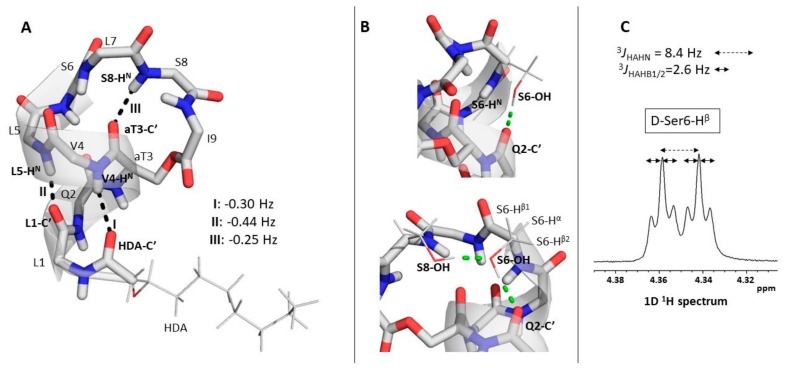
(**A**) The representative structure of VA in acetonitrile (from MD simulation, see further) showing the three experimentally detected backbone hydrogen bonds with black dashed lines and their respective ^3h^*J*_NC′_ values measured in acetonitrile. (**B**) The d-Ser6 O–H···O=C′ d-Gln2 (top) and d-Ser8 O–H···O–C^β^ d-Ser6 hydrogen bonds (bottom) with green dashed lines. The orientation of the d-Ser6 H^α^ and H^β^ atoms is also displayed (bottom). (**C**) The fine structure of the d-Ser6 H^α^ signal in acetonitrile (isotopically non-enriched VA, 700 MHz ^1^H frequency, 298.0 K). The larger and smaller splitting are the result of the ^3^*J*_HNHA_ (Table 1) and the equal ^3^*J*_HAHB1_ = ^3^*J*_HAHB2_, respectively.

In contrast, a cross-peak that would connect d-Ser6 H^N^ and d-Gln2 C′ in the α-helical moiety of the peptide is absent, although it was expected to arise as the associated d-Ser6 N–H···O=C′ d-Gln2 hydrogen-bond can be inferred from the NMR solution structure. This discrepancy was resolved by the MD simulation of VA in acetonitrile which revealed a hydrogen bond from d-Gln2 O=C′ with the side chain hydroxyl group of d-Ser6 instead. This arrangement is apparently further stabilized by an O–H···O–C^β^ hydrogen bond formed by the side chain hydroxyl groups of d-Ser8 (donor) and d-Ser6 (acceptor) (Figure 3B). 

As in the d-Ser6 O–H···O=C′ d-Gln2 interaction an oxygen atom is involved instead of an amide nitrogen, no ^3h^*J*_N*i*C′j_ scalar coupling is associated with it, and therefore, it remains invisible to the long-range HNCO measurement. Nevertheless, the MD simulation suggested that the d-Ser6 side-chain is required to adapt a χ_1_ torsion angle of ~−60° to hold the d-Ser6 O–H···O=C′ d-Gln2 hydrogen bonding. This particular side-chain conformation should generate two equally small ^3^*J*_HAHB_ scalar couplings due to the gauche orientation of both β(C–H) bonds with respect to the α(C–H) one. The fine structure analysis of the d-Ser6 H^α^ and H^β^ signals in the 1D ^1^H-NMR spectrum of the VA compound with natural isotope abundance shows that this is indeed the case, each ^3^*J*_HAHB_ being 2.6 Hz (Figure 3C). As scalar couplings relating to χ_1_ angles were not used during the original structure determination, this specific organization was not generated. While these changes to the solution conformation might be considered rather subtle, they demonstrate the power of hydrogen bond detection to obtain high resolution information on local conformational aspects of CLiP structure, which may go unnoticed using nOe-based structure calculations. The hydrogen bond pattern of VA in DPC solution was also examined by the long-range HNCO approach. Despite the increased line broadenings of the peptide signals, the experiment proved successful and the same three backbone H-bonds seen in acetonitrile were observed (Figure 2B). Similarly, the MD simulation of VA in an explicit DPC + H_2_O environment (detailed later) suggested that permanent d-Ser6 O–H···O=C′ d-Gln2 bonding takes place complemented by the interaction of the d-Ser8 and d-Ser6 side chain hydroxyl groups. The broader resonance line widths obstructed the observation of line splitting in the DPC solution, and thus prevented experimental verification via ^3^*J*_HAHB_ couplings as done in acetonitrile. Nevertheless, the MD simulations and the results of the long-range HNCO experiment indicate that the H-bond pattern of VA observed in acetonitrile is maintained in DPC solution.

In addition to mapping the N–H···O=C′ hydrogen bonding network, valuable information about the “strength” of individual H-bonds can be derived from an analysis of the size of the coupling constants. Indeed, the absolute value of the ^3h^*J*_N*i*C′j_ is a measure of the extent of the associated orbital overlap, a high absolute value indicating a stronger hydrogen bond. Using a quantitative *J*-correlation approach, the ^3h^*J*_N*i*C′j_ value associated with each hydrogen bond was determined to provide a measure of hydrogen bond strength (see Materials and Methods) [44]. Generally, α-helical backbone hydrogen bonds display ^3h^*J*_N*i*C′j_ values of −0.36 ± 0.18 Hz [30] which is in good agreement with the values collected in acetonitrile for the α-helical hydrogen bonds I and II. The smaller absolute value for hydrogen bond III suggests a weaker hydrogen bond strength. (Figure 3A) In DPC solution the value of ^3h^*J*_NiC′j_ for hydrogen bond II (−0.42 Hz) is identical to the one in acetonitrile within the experimental error. Unfortunately, the proximity of the C′ resonance of l-Leu7 to that of d-aThr3 combined with the incomplete suppression of the d-Ser8 N–H to l-Leu7 C′ ^1^*J*_Ni+1C′i_ correlation makes the accurate determination of the ^3h^*J*_NiC′j_ value of hydrogen bond III impossible. Likewise, a similar proximity of the C′ resonances of d-Val4 and the fatty acid (HDA) moiety, combined with the appearance of the intraresidual ^2^*J*_NiC′i_ cross-peak of d-Val4 causes the same problem for hydrogen bond I (Figure 2C). The separation of these overlapping cross-peak pairs could not be achieved neither by the variation of the temperature nor by optimization of the appropriate delay in the long-range HNCO pulse sequence (see Materials and Methods) so as to better remove the effect of the interfering ^1^*J*_NC′_ and ^2^*J*_NC′_ couplings. Besides, as the overlapping cross-peaks always involve the same amide N–H group, the introduction of a third, ^15^N dimension would not resolve them. Therefore, the quantification of the ^3h^*J*_NiC′j_ for hydrogen bonds I and III were not performed.

This notwithstanding, the experimental data obtained by explicit detection of hydrogen bonds independently confirms the conclusion based on comparison of the ^3^*J*_HNHA_ coupling constants in both environments: the backbone conformation of VA in DPC solution is not significantly different from that in acetonitrile. Since the observation of the hydrogen bonds in both conditions requires long, ms to s lifetimes our results also demonstrate the persistent nature of the peptide fold and the general lack of flexibility.

### 2.4. Insertion Depth and Orientation of VA in DPC Micelles Using Paramagnetic Probes

Soluble paramagnetic probes are convenient tools to investigate the insertion and orientation of membrane associated proteins and peptides, as their usage does not require any modifications to be made to the system of interest [45]. The paramagnetic probe enhances the relaxation rates of nearby nuclei in a distance dependent fashion [46]. By monitoring these paramagnetic relaxation enhancements (PREs), information on the location and orientation of the membrane interacting peptide with respect to the water-lipid interface can be inferred. The addition of water soluble Gd(III)DTPA probe to the DPC solution of VA allows to identify moieties that are water exposed or close to the water-lipid interface. Phosphatidylcholine (PC) lipids featuring a nitrosyl radical carrying doxyl group at position 5 or 16 of the hydrocarbon chain were used to probe proximity to the outer rim and the inside of the DPC micelles, respectively, as these lipids co-aggregate with the DPC micelles. PREs on VA in DPC solution were inferred from individual ^1^H–^13^C cross peak intensities in ^1^H–^13^C HSQC spectra. The PRE is calculated as PRE = 1 − (I/I_0_), with I and I_0_ the 2D peak intensities obtained in the presence and absence of a fixed concentration of the paramagnetic probe respectively, implying its value lies between 0 (no PRE) and 1 (full PRE).

**Figure 4:**
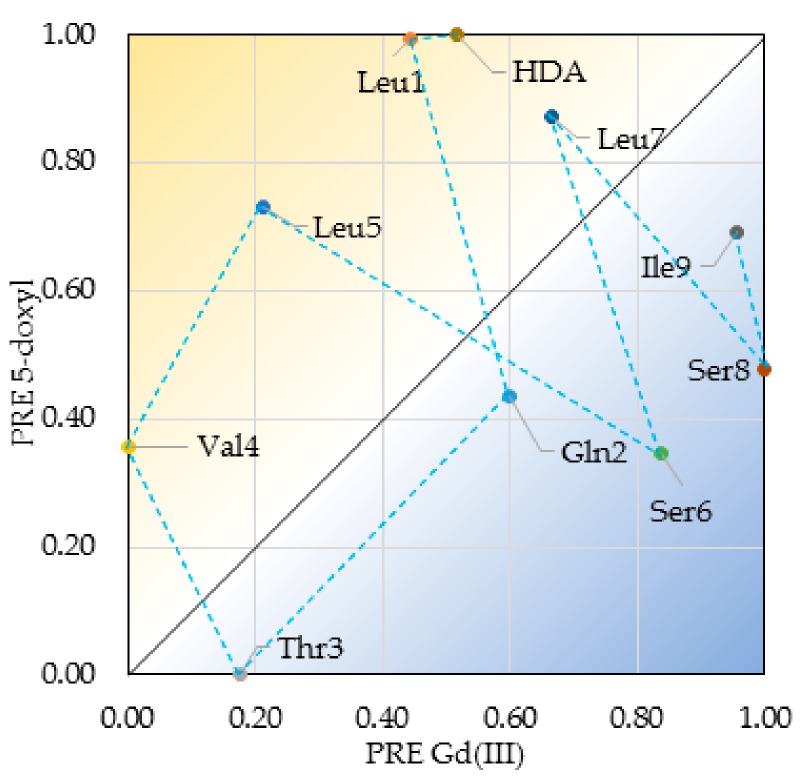
2D scatter plots showing the normalized PRE effect of the lipid-bound 5-doxyl probe (y-axis) vs that of the water soluble Gd(III) probe (x-axis) for the ^1^H–^13^C pairs of both the backbone.

A qualitative inspection of the PREs obtained using the three probes clearly shows that the addition of the doxyl-PC probes resulted in a similar PRE profile along the VA carbon skeleton, while a different PRE profile could be obtained with the Gd(III)DTPA probe (Supplementary Figure S4). Furthermore, in spite of their similar concentration, the PREs caused by 16-doxyl-PC are generally less than 10% of those seen using 5-doxyl-PC, indicating that VA is partitioned closer to the head of the DPC molecules than to the tails in the micelle core. Next, to evaluate the orientation of VA with respect to the water-lipid interface, we wanted to confront the backbone α(C–H) PREs obtained with 5-doxyl-PC probe with the ones obtained with Gd(III)DTPA. For this purpose, each set of PREs was first rescaled so as to put the minimum PRE to 0 and the maximum to 1. This normalization is necessary in order to avoid complications when comparing PREs from different probes due to differences in intrinsic PRE potential and concentration of the probes. These sets of normalized PREs were plotted in a 2D scatter graph as shown in Figure 4. 

Here, residues located on or close to the diagonal indicate a similar exposure to both probes, whereas those located away from the diagonal indicate more exposure to the 5-doxyl-PC in the lipid phase (lower triangle) or the Gd(III)DTPA probe in the water phase (upper triangle). In the resulting graph, the α(C–H) PREs from the hydrophobic residues can be seen as more exposed to 5-doxyl-PC (except for l-Ile9), while those from the hydrophilic residues are clearly more exposed to the Gd(III)DTPA probe. When connecting the data points from the αCH_2_ of HDA to l-Ile9 in the N- to C-direction, a pattern alternating between more 5-doxyl PC and more Gd(III)DTPA exposure emerges that is typical for an α-helix oriented parallel to a water-lipid interphase: Two residues more exposed to the lipid phase alternate with two residues more exposed to the water phase (Figure 4). This alternating pattern breaks down at l-Leu7, which is more exposed to the lipid phase than the water phase, signifying the departure of the backbone from the helix conformation into the loop conformation. In the loop, the α(C–H) of d-Ser8 and l-Ile9 are clearly more water-exposed. A similar graph for the (C–H)-s of methyl groups located at the end of the respective hydrophobic side-chains shows these to be more lipid exposed, while the Ser and Gln methylene groups show their PREs to be dominated by the Gd(III)DTPA probe (Supplementary Figure S5). This analysis firmly establishes that VA is immersed close to the DPC-water interface, with the helix parallel to the interface in a fashion dictated by its amphipathic surface character.

### 2.5. Completing the Picture Using Molecular Modelling in DPC/Water Mixtures

**Figure 5:**
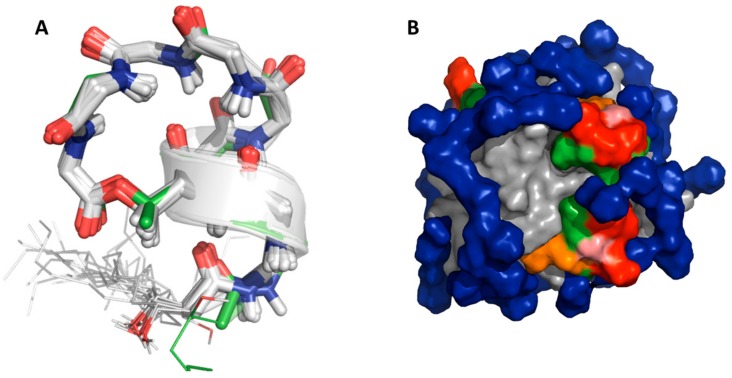
(**A**) Overlay of the 16 peptide representative conformations. The calculated NMR conformation of VA [23] is depicted in green. Side chains are omitted for clarity, except for the fatty acid chain at the C-terminus. (**B**) Representation of a DPC-viscosinamide micelle at the end of the MD simulation (400 ns). The aggregate consists of 63 DPC molecules and 3 VA molecules. DPC and peptide molecules are represented as solvent accessible surfaces. The DPC head groups and alkyl tails are blue and grey, respectively. Polar and hydrophobic amino acids appear red and green respectively.

To assist in the interpretation of all experimental results concerning VA in DPC solution, a 400 ns molecular dynamics simulation (MD) already referred to above was performed starting from an initial configuration where 190 DPC molecules with fully extended chains were placed in a systematic grid, together with 16 VA molecules, and immersed in a water solvent box, to representing a P:L of ~8:100 and a DPC concentration well above the *cmc*. The starting conformation of each VA molecule was taken from the previously calculated solution ensemble in acetonitrile. In the initial phase of the simulation, the DPC molecules quickly aggregated thereby alleviating the unfavorable solvation energetics associated with their long non-polar alkyl chains. The polar choline head groups interact closely and are in contact with the aqueous phase, as can be expected. Strictly speaking, the formation of DPC micelles was not witnessed, as VA molecules were also involved in the initial collapse. In total, 4 larger, stable DPC aggregates containing VA were formed after ~200 ns and persisted until the end of the simulation. As the 16 VA molecules display non-correlated dynamics, their structural evolution was analyzed separately; and a single snapshot structure which represents their time-averaged conformation was chosen for each (see Materials and Methods). These representative conformations are shown superimposed on the solution structure in Figure 5A. 

Excluding the flexible fatty acid part, the backbone RMSD values within the MD ensemble and with respect to the solution structure are 0.13 ± 0.02 Å and 0.44 ± 0.02 Å respectively. These values indicate that the conformation of the individual VA peptides shows limited flexibility during the simulation and remain close to the original solution structure. This is fully in line with the experimental data on ^3^*J*_HNHA_ scalar couplings and the ms to s lifetime of the experimentally detected hydrogen bonds (see above). Interestingly, the mobility of the acyl chain remains high, as seen from the distribution of acyl chain orientations (Figure 5A) and RMSF fluctuations in DPC + H_2_O environment and its comparison to acetonitrile (Supplementary Figure S6). A qualitative impression of the location and orientation of VA with respect to the DPC-water interface is shown in Figure 5B, where one clearly sees that the hydrophilic face of VA is exposed to the lipid head groups and water phase, while the hydrophobic side chain is mostly invisible as it faces the lipid phase on the inside, the center of mass being located close to the transition of the polar head group to the non-polar chain.

**Figure 6:**
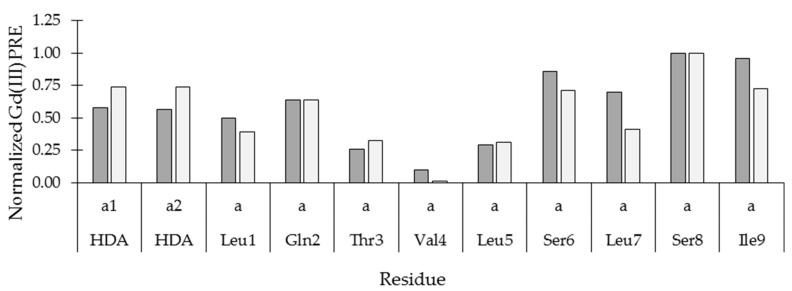
PRE for α(C–H) pair (dark grey) plotted together with normalized number of water molecules in a 5 Å shell around a C^α^ as calculated from the MD simulation (light grey).

A more quantitative analysis is shown in Figure 6, where the normalized PREs induced by Gd(III)DTPA on the α(C–H) along the backbone are compared with the normalized average number of water molecules within a 5 Å sphere centered around the corresponding C^α^ atom in the 16 individual VA trajectories. The agreement is good to excellent, with d-Val4 located in the middle of the hydrophobic face oriented towards the micelles’ center being least exposed and d-Ser8 being most exposed to the water phase. 

**Figure 7:**
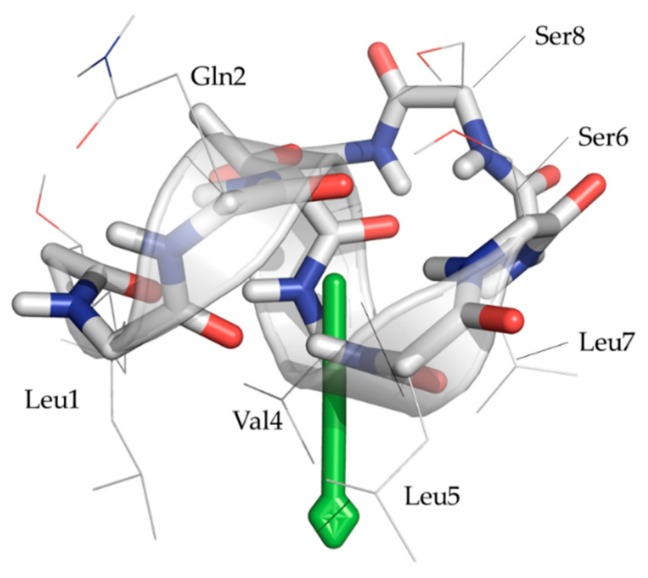
Representative VA conformation at the DPC-water interface, showing the hydrophobic moment as a green vector arrow.

When the hydrophobic moment of a representative VA conformation is calculated and used for orientation the aforementioned residues are indeed seen at opposing ends along the hydrophobic moment (Figure 7). 

In addition, the moment is close to orthogonal to the helix’ axis. If the hydrophobic moment is assumed to point towards the core of the lipid phase, this brings the amphipathic helix in an orientation parallel to the DPC-water interface. Closer inspection of the fatty acid moieties during the simulations shows that the acyl chain penetrates deeper inside the micelle while the peptide chain is located at the interfacial region and does not penetrate into the hydrophobic micelle core. All these observations are in line with the experimental results described above, including the observation that in the presence of 16-doxyl-PC, the methyl group at the end of the VA acyl chain experiences the largest PRE effect of all α(C–H). 

## 3. Discussion

We have provided the first analysis of the interaction of a CLiP, viscosinamide (VA) with zwitterionic DPC as membrane mimicking model system using liquid state NMR spectroscopy and molecular dynamics simulations. This combined approach allowed to obtain detailed information concerning the location and orientation of a neutral viscosin group member at a water-lipid interface.

Using diffusion-ordered NMR spectroscopy, it was found that incorporation of VA into the DPC micelles is close to 100%. Characteristic nOe contacts indicated that the α_L_-helix observed in free, monomeric state of VA remained present when the peptide is transferred to a membrane mimicking environment consisting of DPC micelles. Moreover, isotope enrichment was introduced allowing to extend the extraction of ^3^*J*_HNHα_ coupling constants to VA in DPC solution. It also provided direct access to the detection of long-lived hydrogen bonds involving the backbone amide groups in different environments, showing these are maintained throughout. From these results, we conclude that no conformational changes occur for VA, despite the peptide–micelle interactions. This is clearly different from the behavior of the majority of cationic antimicrobial peptides, which are partly or completely unstructured in solution and only adopt a specific fold as part of the peptide-membrane interaction process [47,48]. As a result, the enthalpic and entropic changes involved in the peptide–membrane interactions of viscosin group CLiPs can be expected to be quite distinct from those of cAMP’s and merit further biophysical investigation. Our results assist in these, in that any such investigations (using i.e. CD, ITC, SPR, fluorescence spectroscopy) of the peptide-membrane interactions can use the three-dimensional structure of these compounds obtained using NMR in (organic) solvents or from crystals as a valid model for analysis and interpretation. 

Using paramagnetic relaxation enhancement, the orientation and insertion depth of VA was determined. The attenuation of NMR signals of VA in the presence of various paramagnetic probes is in good agreement with the amphipathicity of the molecules whereby the CLiP is inserted with its helix parallel to the water-lipid interface, close to the transition between polar head group and non-polar chain, whereas the fatty acid residue displays a somewhat deeper insertion in the micelle core. The hydrophobic residues are directed towards the micelle while the polar residues point towards the aqueous phase. This was independently confirmed using a molecular dynamics approach. Moreover, the determined orientation of the individual VA molecules on a membrane bilayer matches well with the orientation of the hydrophobic vector of the CLiP. Thus, if sufficient VA molecules are present, we assume that they may cover the top layer of a membrane bilayer. Nonetheless, we also expect that at concentrations well below the onset of permeabilization, CLiPs such as VA could already significantly perturb the order/fluidity/structure of the bilayer, which may affect its function or that of other components in it. Our results, using the DPC:water binary system as a model, should prove useful to further investigate the biological effects resulting from the interaction of CLiPs with prokaryotic and eukaryotic membranes. 

## 4. Materials and Methods

VA at natural isotope abundance was produced by *Pseudomonas* sp. DR54 and was extracted, purified and characterized as previously described [23]. The uniformly ^15^N-enriched VA was produced as follows: bacterial cells of *Pseudomonas* sp. DR54 strain were transferred into 4 × 5 mL King’s Broth medium (Difco Laboratories, Sparks, MD, USA) and grown for 1 day under equilibrated conditions (28 °C, 150 rpm shaking frequency). Afterwards, each of the 5 mL cultures were transferred into a 2L Erlenmeyer flask containing 400 mL of M9 minimal medium in which ^15^N-enriched ammonium chloride was the sole nitrogen source. Following an additional 24 h cultivation under minimal conditions (28 °C, 150 rpm shaking frequency), the culture was centrifuged to separate the supernatant from the solid cells. The volume of the medium was reduced to 250 mL using rotary evaporation, and then extracted three times using an equal volume of ethyl acetate containing 1% formic acid. After rotary evaporation, the resulting crude was stored in the freezer. The cell pellet that remained after centrifugation was subjected to 3 cycles of freeze-thawing in ethyl acetate containing 1% formic acid. After rotary evaporation, the crude was successively subjected to a solvent extraction using methyl-tert-butyl ether, water, chloroform and acetonitrile. As the NMR spectra of the extracted compound from the cells reflected its high purity, further HPLC purification was not performed. Successful incorporation of the ^15^N isotopes at levels above 95% was easily demonstrated from the integral of the ^15^N satellites relative to the central resonance in the 1D ^1^H NMR spectra (Supplementary Figure S3). To prove the chemical identity of the ^15^N-enriched and the VA, the ^1^H-^13^C gHSQC spectra of both compounds were confronted and showed high level of similarity. (Supplementary Figure S7) The bacterial production and extraction of the uniformly ^13^C- and ^15^N-enriched version of VA followed the same steps as of the uniformly ^15^N isotope-enriched version by supplementing the M9 minimal medium with uniformly ^13^C-enriched d-glucose (U-^13^C-d-glucose) as sole carbon source. Subsequent purification was performed as described for the isotopically non-enriched VA. In total ~35 mg of the main CLiP compound could be collected. The ESI-MS analysis of this compound displayed pseudomolecular ions at the *m*/*z* value of 1189.7 most abundantly (Supplementary Figure S8). Compared to the monoisotopic value of [VA + H]^+^ (1125.7 *m*/*z*) [23] this implies a +64 Da change in molecular mass what can be explained by the efficient replacement of the ^12^C by ^13^C, and the ^14^N by ^15^N isotopes in VA (C_54_H_96_N_10_O_15_). In addition, the matching ^1^H–^13^C (constant-time) gHSQC spectra of the ^13^C-,^15^N-enriched and the isotopically non-enriched VA demonstrated their chemical identity (Supplementary Figure S7). 

For the bacterial growth experiments ^15^NH_4_Cl and the U-^13^C-d-glucose were purchased from Cambridge Isotope Laboratories (Tewksbury, MA, USA). For NMR measurements deuterated solvents were purchased from Eurisotop (Saint-Aubin, France). Dodecylphosphocholine-d38 (DPC), 1-palmitoyl-2-stearoyl-(5-doxyl)-sn-glycero-3-phosphocholine and 1palmitoyl-2-stearoyl-(16-doxyl)-sn-glycero-3-phosphocholine were purchased from Avanti Polar Lipids (Alabaster, AL, USA). The water-soluble gadolinium(III) diethylenetriaminepentaacetic acid (Gd(III)DTPA; Magnevist^®^) was acquired from Sigma-Aldrich (Saint Louis, MO, USA). High precision 5 mm NMR tubes (Norell Inc., Morganton, NC, USA) were used for all NMR experiments. 

The NMR measurements were performed on either an Avance III spectrometer operating at a respective ^1^H and ^13^C frequency of 500.13 MHz and 125.76 MHz equipped with a BBI-Z probe or an Avance II spectrometer (Bruker, city, state abbrev if USA, country) operating at a respective ^1^H and ^13^C frequency of 700.13 MHz and 176.05 MHz and equipped with either a ^1^H, ^13^C, ^15^N TXI-Z probe or 5 mm Prodigy TCI probe. The sample temperature was set to 298.0 K in most cases. However, the HNHA and the triple resonance experiments were conducted on the isotope-enriched VA compounds in DPC solution at 310.0 K to reduce signal broadenings.

Standard pulse sequences as present in the Bruker library were used throughout, unless stated otherwise. 2D spectra were measured for the characterization of the isotopically-enriched VA compounds including ^1^H–^1^H TOCSY with a 90 ms MLEV-17 spinlock, constant-time ^1^H–^13^C gHSQC, ^1^H–^15^N HSQC, HNCO, HNCA and HN(CO)CA. The direct ^1^H dimension was created by sampling 2048 data points with 11 ppm spectral width in all cases. For the triple resonance experiments the acquisition of the ^15^N dimension was omitted. The indirect ^1^H or ^13^C dimensions of the ^1^H–^1^H TOCSY and the ^1^H–^13^C gHSQC experiments were recorded by sampling 512 data points with the spectral width of 11 ppm and 110 ppm, respectively. The indirect ^15^N or ^13^C dimensions of the ^1^H–^15^N HSQC and the triple resonance experiments were recorded by sampling 128 data points with a spectral width varied between 10 ppm and 110 ppm, depending on the experiment. For 2D processing, the spectra were zero filled to a 2048 × 2048 real data matrix. Before Fourier transformation, all spectra were multiplied with a squared cosine bell function in both dimensions. When applicable, excitation sculpting or a selective 90° water flip-back pulse was used to suppress the H_2_O signal. 

PFGSTE translational diffusion measurements were performed by using convection compensated sequences. Either the standard Bruker double stimulated echo with bipolar gradients [49] or a double stimulated echo with monopolar gradients with an extended phase cycle [50] was used. The diffusion encoding/decoding gradients were varied linearly between 2% and 95% of their maximum output over 32 increments. The duration of these gradients and the diffusion delay time were chosen so that at the highest gradient strength the intensity of the signals of interest was decreased to at least 10% of the intensity at the lowest gradient strength. The obtained intensity decays were fitted to the appropriate Stejskal–Tanner equation [51] using an in-house Matlab script.

The 3D HNHA experiments were recorded on uniformly ^15^N-enriched VA samples using identical acquisition and processing parameter set. The ^15^N-VA was dissolved up to 1.5 mM in acetonitrile, or up to 0.5 mM in 100 mM DPC/90%H_2_O + 10%D_2_O, pH = 7.4. The amide H^N^ and H^α^ regions of the spectra were already sparse even if the acquisition of the ^15^N dimension was omitted and the experiment was run in 2D fashion by recording the H^α^–H^N^ plane within an hour. To fully resolve the limited number of peak overlaps then, the ^15^N dimension could be acquired with a restricted number of time domain points. The direct H^N^ (*t*_3_), indirect H^α^ (*t*_2_) and indirect ^15^N (*t*_1_) dimensions were recorded by sampling 2048, 64 and 8, data points, with acquisition times of 121.7, 3.8 and 1.8 ms, respectively. The experiment was repeated 32 times per each indirect time domain point with a relaxation delay of 1 s. The total experimental time was 5.5 h. For 3D processing the time domain data were zero filled up to a 2048 × 256 × 64 real data matrix. Before Fourier transformation, the time domain data were multiplied with a squared cosine bell function along all dimensions, and one order of forward linear prediction was also applied in *t*_2_ and *t*_1_. The absorptive part of the resulting 3D spectrum was phase corrected along *F*_3_ where small baseline distortions were also corrected for by an automatic 5th order baseline correction routine. The ^3^*J*_HNHA_ values per amino acid can be quantified in a J-correlation approach. That is, in the *F*_2_ strips taken at the respective ^15^N–H^N^ resonance position of each amino acid, the intensity ratio or—provided that the line shapes are identical—the integral ratio of the H^N^–H^α^ cross-peak to the respective H^N^ diagonal peak presents a measure for the ^3^*J*_HNHA_:

(1) ScrossSdiag=−tan2π JHNHA32ζ

in Equation (1) ζ ~ $\frac{1}{4J_{\mathrm{HNHA}}}$ is the length of the de- and rephasing delays during which the *J*_HNHA_ is active. The ζ parameter was set to the default 13.05 ms and the S_cross_/S_diag_ was assessed by manual 2D integration. The amino acid-wise comparison of the ^3^*J*_HNHA_ (AcN) values extracted from the HNHA experiment and the ones read from the 1D ^1^H spectrum of the natural VA shows excellent correlation without the sign of any systematic discrepancy (Table 1 and Supplementary Figure S9). Such comparison of the ^3^*J*_HNHA_ (AcN) and the ^3^*J*_HNHA_ (DPC) values, both measured by the HNHA experiment, still displays great linear correlation. In this case, however, the ^3^*J*_HNHA_ (AcN) of the amino acids within the macrocycle appeared slightly (0.1–0.4 Hz) but consistently larger (Supplementary Table S4 and Supplementary Figure S9). This suggests the systematic underestimation of the actual ^3^*J*_HNHA_ (DPC), an effect that originates from enhanced relaxation due to the hindered tumbling of VA upon its interaction with the DPC micelles. The set of actual ^3^*J*_HNHA_ (DPC) values (Table 1) could then be obtained by multiplying each ^3^*J*_HNHA_ (DPC) values calculated from the HNHA spectrum by a uniform factor of 1.043. 

The long-range HNCO experiments were recorded on uniformly ^13^C-, and ^15^N-enriched VA dissolved up to 2.81 mM in acetonitrile, or up to 5.8 mM in 150 mM DPC/90%H_2_O + 10%D_2_O, pH = 7.4. For both samples, the direct H^N^ (*t*_3_), indirect ^15^N (*t*_2_) and indirect ^13^C′ (*t*_1_) dimensions were recorded by sampling 2048, 1 and 128, data points, respectively, resulting in the acquisition of a 2D H^N^–C′ plane without the ^15^N dimension. The acquisition time was uniform in *t*3 (104.4 ms), and *t*2 (0.2 ms) while in *t*1 it was slightly longer in DPC solution (42.3 ms) than in acetonitrile (30.3 ms) to increase the resolution. The experiments were repeated 256 times per each indirect time domain point with a relaxation delay of 1.2 s, yielding a total experiment time of ~15 h. For 2D processing the time domain data were zero filled up to a 2048 × 512 real data matrix. Before Fourier transformation, the time domain data were multiplied with a squared cosine bell function along all dimensions, and one order of forward linear prediction was also applied in *t*_1_. The absorptive part of the resulting 2D spectrum was phase corrected along *F*_3_ where small baseline distortions were also corrected for by an automatic 5th order baseline correction routine. The ^3h^*J*_NC′_ values of the detected hydrogen-bonds can be quantified in a J-correlation approach. That is, the intensity of a hydrogen bond cross-peak (I_cross_) has to be compared to the one of its ‘reference’ peak (I_ref_) that is obtained in a separately recorded experiment. The long-range HNCO experiment employs quasi-simultaneous ^15^N and ^13^C′ 180^o^ pulses in the ^15^N→^13^C′ INEPT (or ^13^C′→^15^N reverse-INEPT) modules which by default operates with the total evolution period of 2T = 133.3 ms to satisfy the 2T ~ 2JNC′1 approximation. Thus, the effect of ^1^*J*_NC′_-s does not evolve, while the effect of weaker couplings such as ^3h^*J*_NC′_ can be detected. In the reference HNCO however, the ^13^C′ 180^o^ pulse is delayed with respect to the ^15^N 180° pulse with ε = 16.6 ms ~ 24 JNC′1 while the total evolution period stays the chosen value of 2T. In this case therefore, reference peaks resulting from the ^1^*J*_NC′_ coupling show up exclusively, and the effect of the weaker *J*_NC′_ couplings is obliterated. Apart from the introduction of the ε delay, the respective reference and the long-range HNCO experiments have to be recorded with identical acquisition parameter set for the quantification of ^3h^*J*_NC_ values. The corresponding reference and hydrogen bond cross-peak appear at the same resonance frequency along the H^N^ dimension in the two spectra. Assuming that ^1^*J*_NC′_ = −15 Hz, the value of ^3h^*J*_NC′_ can be derived from the following formula: [43]:

(2) IcrossIref=sin2π J3hNC′2Tcos2π J3hNC′2T−2ε

Paramagnetic relaxation enhancement at various locations in the DPC micelle was measured by means of ^1^H–^13^C gHSQC spectra with varying amounts of PRE probes. These probes were either Gd(III)DTPA, 1palmitoyl-2-stearoyl-(5-doxyl)-sn-glycero-3-phosphocholine (hereafter referred to as 5doxyl) and 1palmitoyl-2-stearoyl-(16-doxyl)-sn-glycero-3-phosphocholine (hereafter referred to as 16doxyl). The water-soluble Gd PRE probe was added in different concentrations varying between 0 mM, 0.5 mM, 1.0 mM, 1.5 mM, 2.0 mM, 2.5 mM, 3.0 mM and 3.5 mM) to a DPC sample (100 mM) containing 7.0 mM viscosinamide A. For quantification, the measurements at 3mM GD(III)DTPA were found to represent the optimal signal quenching for all resonances. Additionally, different DPC samples (100 mM) were prepared incorporating either 2.1 mM 5-doxyl or 16-doxyl probes, each with 6.6 mM VA. 

To quantify the signal attenuation caused by the presence of the PRE probes, a PRE factor was calculated as:

(3) $$\mathrm{PRE}=1-\frac{I}{I_{0}}$$

where by I_0_ represents the signal intensity in the absence of the probes, and I denotes the signal intensity at the specific PRE probe concentration used. The PRE factor is then normalized by setting the maximum PRE factor to 100% and the minimum to 0% for each of the probes that were used. This allows for a broad distribution between different PRE factors. The aim of the normalization is to compensate for the differences in intrinsic PRE potential and concentration of the probes.

To assist in visualizing but also to validate the orientation and insertion depth of the lipopeptides derived from experimental data, a molecular dynamics simulation of a DPC micelle composed of 191 monomers were carried out in explicit water using a “self-assembling” approach [52]. Specifically, 191 DPC molecules were oriented in a grid, together with 16 VA molecules. This systems consisted of a periodic cubic box filled with 38919 TIP3P water molecules and 10 K^+^ and 10 Cl^−^ ions. This corresponds to a solution of 22 mM VA in 272 mM DPC, which gives a slightly higher peptide:lipid ratio than the experimental value. The peptide part of the viscosinamide A molecules was parametrized using the Amber ff14SB force field, while the fatty acid tail of the peptide and the DPC molecules were parametrized using the LIPID14 force field [53]. An additional simulation of a single VA molecule in a united-atom acetonitrile solvent box was also carried out using identical parameters. Both simulations represent a 400 ns simulation time. Bonds lengths involving hydrogen were held fixed with the SHAKE algorithm, so that a time step of 2 fs could be used [54]. Temperature scaling was performed using Langevin dynamics with a collision frequency of 1.0 ps^−1^ while electrostatic interactions were computed using the particle mesh Ewald summation. Non-bonded interactions up to 9 Å were computed. Initially, the system was stabilized by using a two-step energy minimization. In the first stage, the solute molecules are fixed with a strong positional restraint (500 kJ mol^−1^) and only the positions of the solvent and ions are submitted to energy minimization. Subsequently, in the second stage, the entire system is minimized without the application of restraints. Then, the system is allowed to heat up from 0 K to 300 K while a weak potential energy restraint (10 kJ mol^−1^) is applied on the solute molecules. This step is done at constant volume to reduce inaccuracies in the beginning of the simulation, when the temperature is very low. Finally, production runs were performed using constant pressure dynamics with isotropic position scaling. All MD simulations were run on a single NVidia GTX680 or GTX780 GPU using the GPU implementation of pmemd provided in the AMBER14 simulation package. [55] The calculated trajectories were visualized using VMD 1.9.1 [56] or Pymol v1.3 while ptraj or cpptraj programs [57] (provided with the AMBER software) were used for in-depth analysis. A cluster analysis protocol was used to select the 3D conformation which ideally reflects the time-averaged position of the atomic coordinates using the built-in DBSCAN [58] algorithm of cpptraj. Thus, according to the coordinate RMSD of a selected set of atoms it was possible to distinguish a cluster of converged conformations from less similar structures (or ‘noise’) that appeared during the trajectory. Additionally, the algorithm determines the snapshot conformation which’s atomic coordinate RMSD is minimal with respect to the centroid of the dominant cluster. This conformation is then used to represent the MD trajectory. For the analysis of the aforementioned trajectories, we defined the coordinate RMSD of all the H^N^, C′, C^α^, N, amide O and Ser side chain OH atoms as clustering criterion. Hydrophobicity vectors were calculated using the web application 3D-HM. [59]

## Figures and Tables

**Table 1:** Comparison of ^3^*J*_HNHA_ coupling constants and corresponding angles measured in acetonitrile and DPC solution.

Amino Acid	^3^*J*_HNHA_ * in Acetonitrile		^3^*J*_HNHA_ * in DPC Solution	HNHA Δ^3^*J*_HNHA_ [Hz]	Calculated φ ^a^		
	1D ^1^H [Hz]	HNHA [Hz]	HNHA [Hz]		φ_ACN_ [°]	φ_DPC_ [°]	Δ [°]
l-Leu1	5.8	6.1	6.6	0.5	33.3	39.8	6.5
d-Gln2	4.2	4.4	3.9	−0.5	61.8	57.6	−4.2
d-aThr3	7.6	7.5	7.6	0.1	84.9	85.3	0.38
d-Val4	5.9	5.9	5.7	−0.2	73.0	71.7	−1.4
l-Leu5	6.7	6.7	6.5	−0.2	78.2	80.8	2.6
d-Ser6	8.4	8.3	8.5	0.2	91.1	93.1	2.0
l-Leu7	5.9	5.9	5.8	−0.1	−72.7	−72.4	0.35
d-Ser8	9.0	8.8	9.0	0.1	96.3	97.5	1.2
l-Ile9	10.2	10.2	10.1	−0.1	x	x	x

^a^ Calculated using the Karplus equation derived from X-ray and NMR data in [42]. Since l-Ile9 features an ester rather than an amide bond, no appropriate Karplus equation is available to convert ^3^*J* into the associated torsion angle. * The values are rounded up to the first decimal.

## Supplementary Materials

The following are available online: **Figure S1**: Step-wise addition of viscosinamide A to a 100 mM DPC solution showed no evolution in ^1^H chemical shift or resonance line width. **Figure S2**: 1D ^1^H spectrum of A) viscosinamide A in 100 mM DPC solution, B) viscosinamide A in acetonitrile. **Figure S3**: A) 1D ^1^H spectrum of ^15^N-enriched viscosinamide A in acetonitrile; B) zoom of the amide region of A). **Figure S4**: Paramagnetic relaxation factors of all viscosinamide A resonances in DPC solution with the presence of A) water-soluble Gd(III)DTPA, B) lipid-bound 5-doxyl and C) lipid-bound 16-doxyl PRE probes. **Figure S5**: PRE XY plots of viscosinamide A side chains in a membrane environment. **Figure S6**: RMSF fluctuations of viscosinamide A during the MD simulations in explicit acetonitrile and DPC + H_2_O environment. **Figure S7**: Overlay of the ‘α-regions’ from the ^1^H-^13^C gHSQC spectra in of the isotopically non-enriched VA (blue) and A) the ^15^N-enriched VA (red) or B) the ^15^N-/^13^C-enriched VA (red). The spectra were recorded in acetonitrile at 700 MHz ^1^H frequency and 298.0 K sample temperature. **Figure S8**: A) The ESI-MS total ion chromatogram (TIC) of ^13^C,^15^N-enriched VA. B) The 1170–1200 *m*/*z* region of the TIC shown in A. The abundance of the 1189.7 *m*/*z* value proves that the isotope enrichment took place with high efficiency. **Figure S9**: Pairwise comparison of ^3^*J*_HNHA_ values per amino acid A) in acetonitrile read from the 1D ^1^H spectrum vs measured by the HNHA method; or B) measured by HNHA method in acetonitrile vs in DPC solution. Least square linear regression was used to fit a linear to the points of the graphs by fixing the intercept to 0. The R^2^ and the slope of the fitted linear are indicated. As in case of B) the points of d-Leu1 and d-Gln2 (marked with red) somewhat deviate from the linear that can be fitted to the points of the residues involved in the macrocycle, they were omitted from the linear regression analysis. **Table S1**: Average φ angles per amino acid over the trajectories of 400 ns-long molecular dynamics simulations in explicit acetonitrile and DPC+H_2_O environment. **Table S2**: Chemical shifts of viscosinamide A (natural isotope abundance) in acetonitrile-d3 at 298.0 K. **Table S3**: Chemical shifts of viscosinamide A (natural isotope abundance) in 100 mM DPC-d38 at 298.0 K. **Table S4**: The ^3^*J*_HNHA_ values obtained in acetonitrile and in DPC solution.
