# Supplementary material for: Conformation and Dynamics of the Cyclic Lipopeptide Viscosinamide at the Water-Lipid Interface
Source: Molecules. 2019 Jun 17;24(12):2257. doi: 10.3390/molecules24122257 (PMC6630293; doi:10.3390/molecules24122257)
Supplement: Supplementary file 1 [file molecules-24-02257-s001.pdf]

Supplementary information

Conformation and Dynamics of the Cyclic Lipopeptide Viscosinamide at the Water-Lipid Interface

Niels Geudens [1,†], Benjámin Kovács [1,†], Davy Sinnaeve [1,§], Feyisara Eyiwumi Oni [2], Monica Höfte [2] and José C. Martins [1,*]

[1] NMR and Structural Analysis Unit, Department of Organic and Macromolecular Chemistry, Ghent University; Campus Sterre, S4, Krijgslaan 281, B-9000 Gent, Belgium; niels.geudens@ugent.be (N.G.); benjamin.kovacs@ugent.be (B.K.); davy.sinnaeve@ugent.be (D.S.)

[2] Laboratory of Phytopathology, Department of Plants and Crops, Ghent University, Coupure Links 653, B-9000 Gent, Belgium; FeyisaraEyiwumi.Olorunleke@UGent.be (F.E.O.); monica.hofte@ugent.be (M.H.)

§ Current Address: CNRS, UMR 8576 Unité de Glycobiologie Structurale et Fonctionnelle, Université de Lille, 59000 Lille, France

* Correspondence: jose.martins@ugent.be; Tel.: +32-9-264-4469

† These authors contributed equally to the work.

Figures

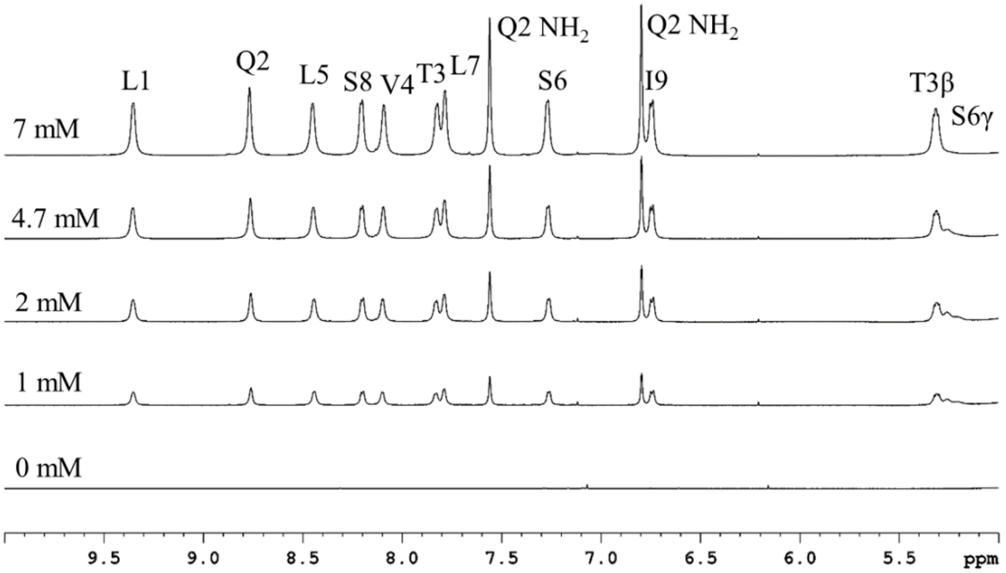

Figure 1. Step-wise addition of viscosinamide A to a 100 mM DPC solution showed no evolution in ^{1}H chemical shift or resonance line width.

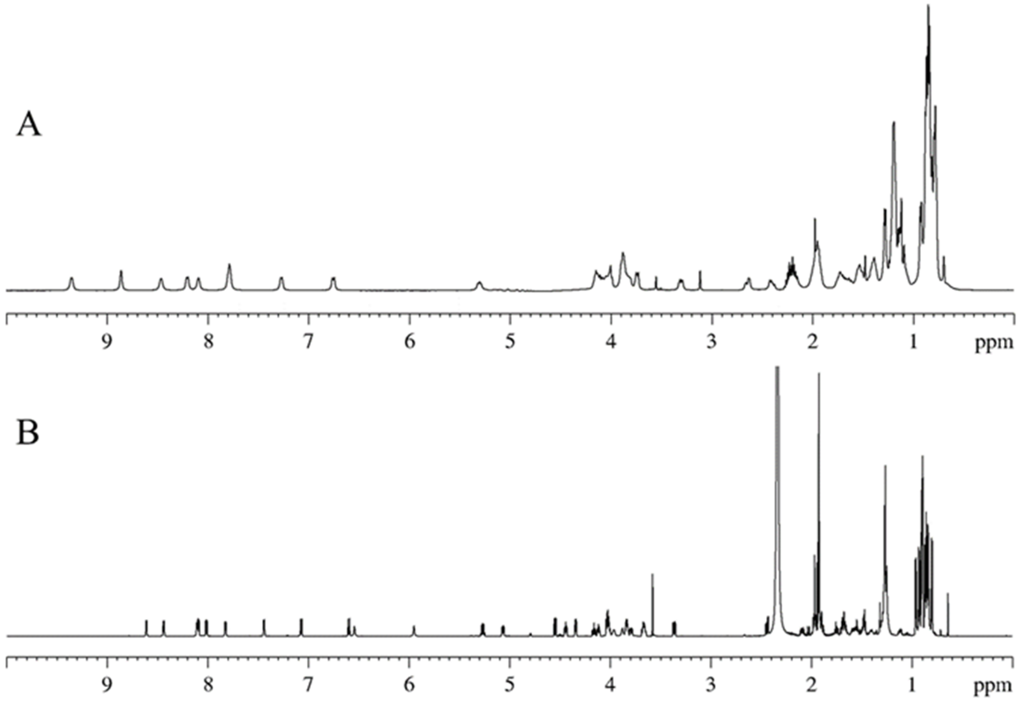

Figure 2. 1D ^{1}H spectrum of A) viscosinamide A in 100 mM DPC solution, B) viscosinamide A in acetonitrile.

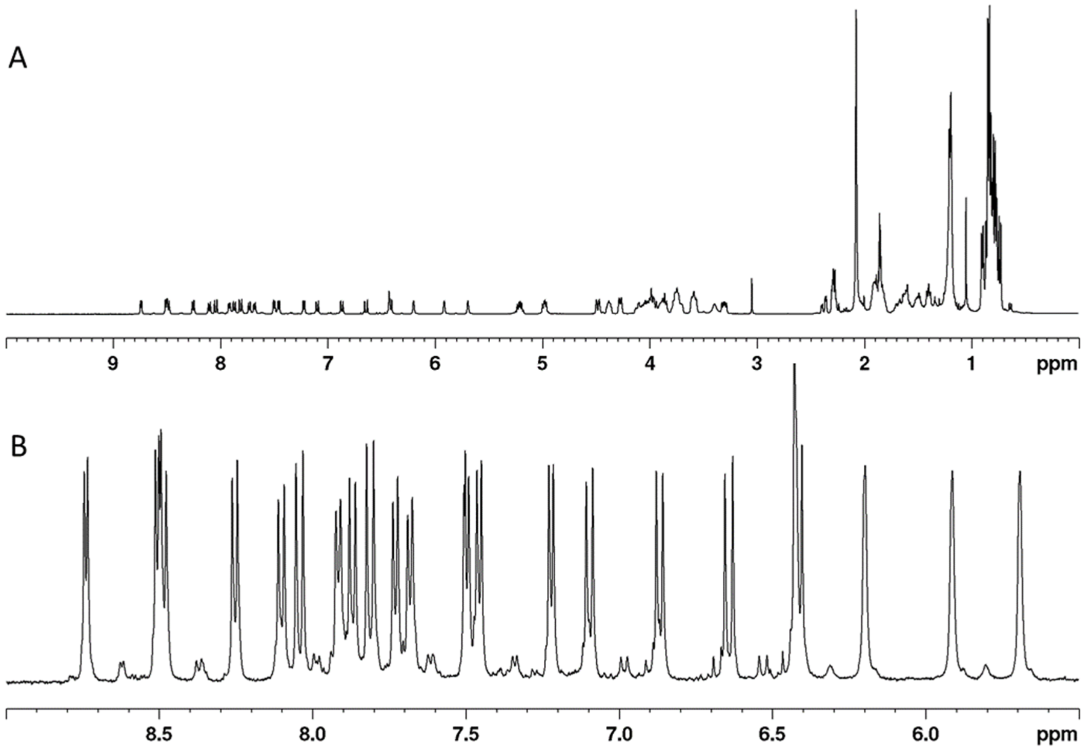

Figure 3. A) 1D ^{1}H spectrum of ^{15}N-enriched viscosinamide A in acetonitrile; **B**) zoom of the amide region of A).

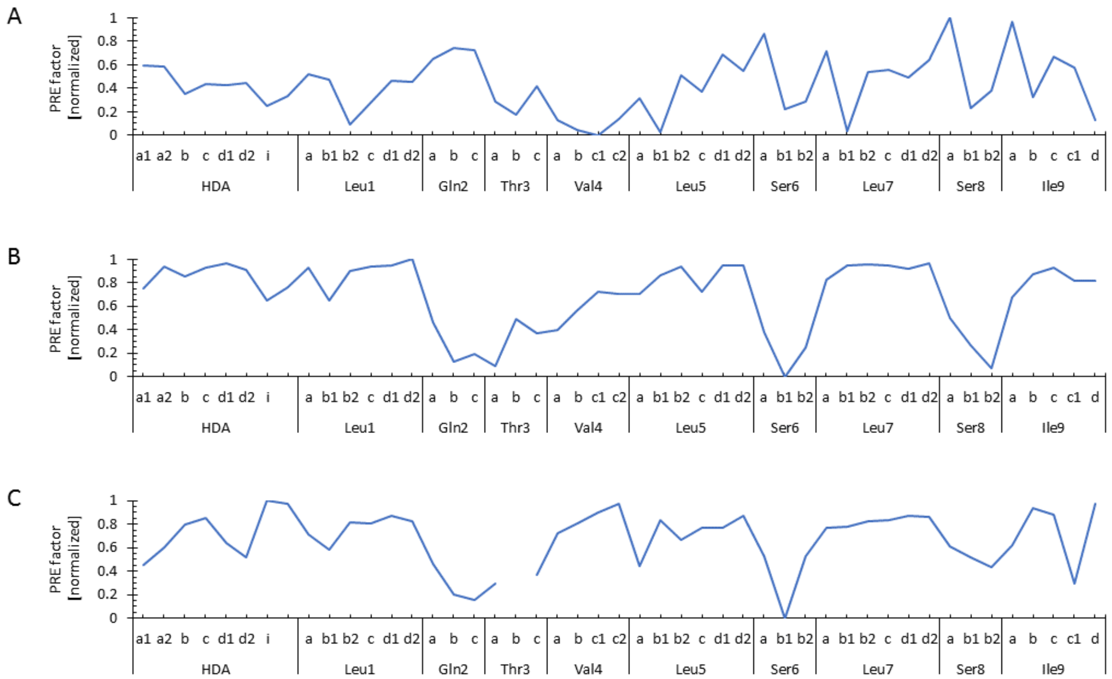

Figure 4. Paramagnetic relaxation factors of all viscosinamide A resonances in DPC solution with the presence of **A**) water-soluble Gd(III)DTPA, **B**) lipid-bound 5-doxyl and **C**) lipid-bound 16-doxyl PRE probes.

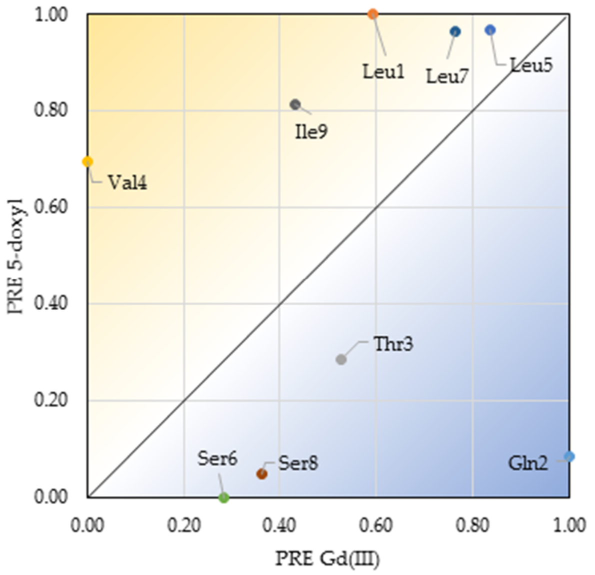

Figure S1: PRE XY plots of viscosinamide A side chains in a membrane environment.

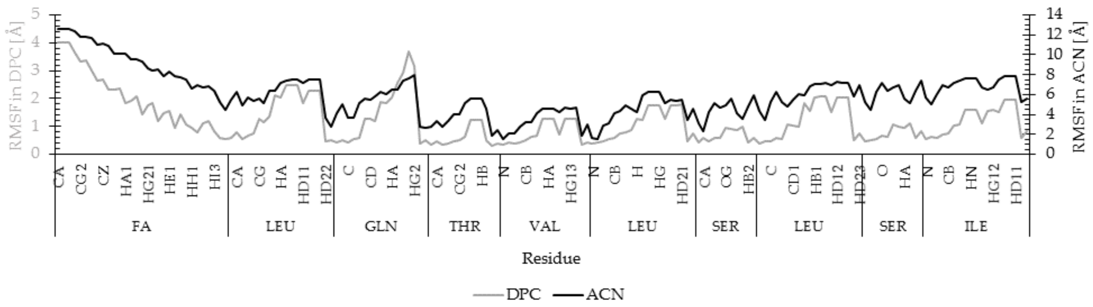

Figure 6. RMSF fluctuations of viscosinamide A during the MD simulations in explicit acetonitrile and DPC+H_2O environment.

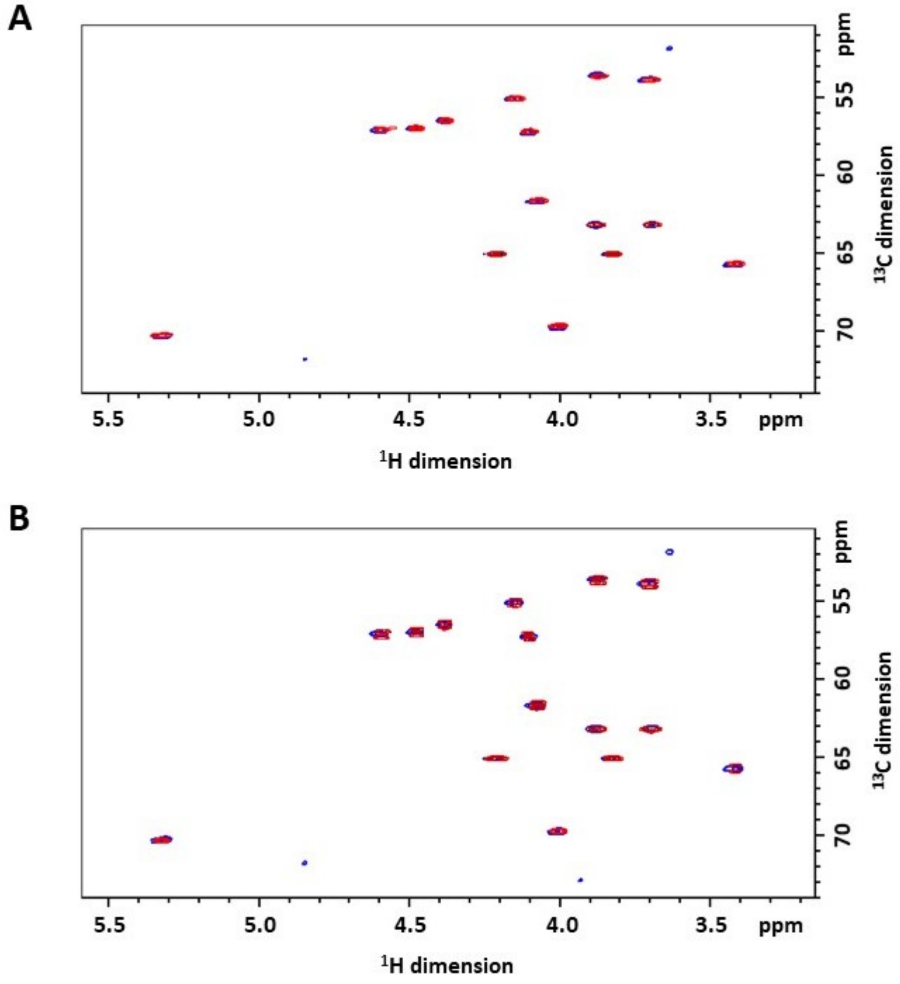

Figure S2: Overlay of the 'α-regions' from the ^{1}H-^{13}C gHSQC spectra in of the isotopically non-enriched VA (blue) and **A**) the ^{15}N-enriched VA (red) or **B**) the ^{15}N-/^{13}C-enriched VA (red). The spectra were recorded in acetonitrile at 700 MHz ^{1}H frequency and 298.0 K sample temperature.

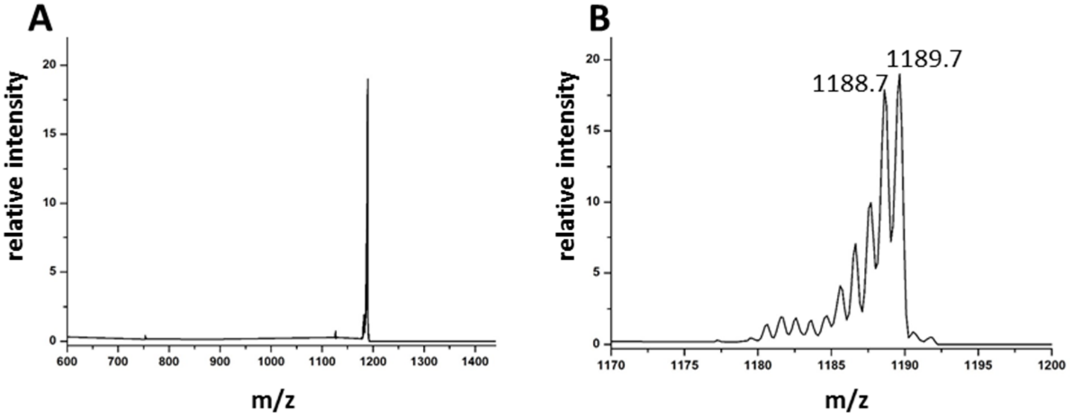

Figure S8: A) The ESI-MS total ion chromatogram (TIC) of ^{13}C,^{15}N-enriched VA. B) The 1170 – 1200 *m*/*z* region of the TIC shown in A. The abundance of the 1189.7 *m*/*z* value proves that the isotope enrichment took place with high efficiency.

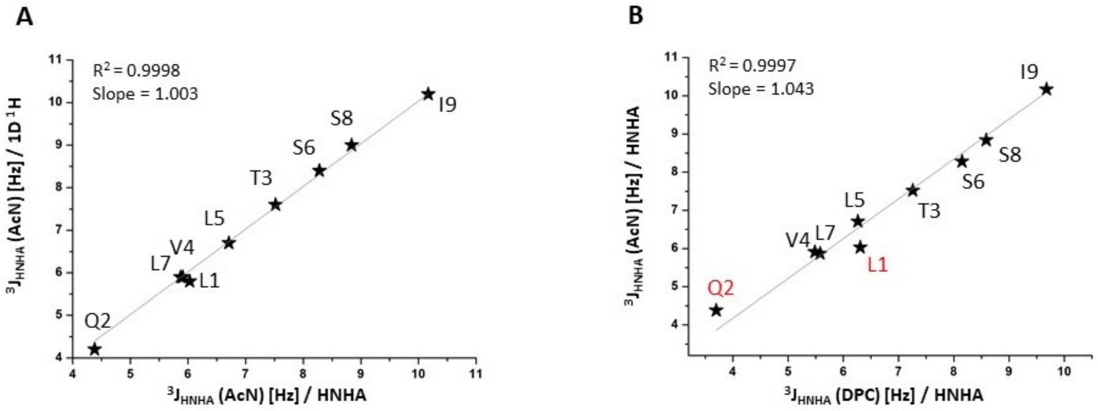

Figure S9: Pairwise comparison of $^3J_{HNHA}$ values per amino acid A) in acetonitrile read from the 1D ^1H spectrum vs measured by the HNHA method; or B) measured by HNHA method in acetonitrile vs in DPC solution. Least square linear regression was used to fit a linear to the points of the graphs by fixing the intercept to 0. The R^2 and the slope of the fitted linear are indicated. As in case of B) the points of D-Leu1 and D-Gln2 (marked with red) somewhat deviate from the linear that can be fitted to the points of the residues involved in the macrocycle, they were omitted from the linear regression analysis.

Tables

Table S1: Average φ angles per amino acid over the trajectories of 400 ns-long molecular dynamics simulations in explicit acetonitrile and DPC+H_2O environment.

Amino acid	Simulated average φ values*/°		
	Acetonitrile medium	DPC/H_2O medium	φ(DPC/H_2O) – φ(AcN) /°
L-Leu1	19.6	46.3	26.6
D-Gln2	64.0	66.1	2.1
D-aThr3	66.7	67.7	0.9
D-Val4	67.4	64.1	-3.3
L-Leu5	55.3	56.2	0.9
D-Ser6	118.4	129.3	10.8
L-Leu7	-68.4	-66.6	1.9
D-Ser8	92.9	98.7	5.7
L-Ile9	-101.5	-85.6	15.9

*The φ values were assessed for each amino acid including the converged snapshots of the simulated trajectories, and their respective averages were taken (see Materials and Methods). Non-converged snapshots were neglected for the averaging.

Table S2: Chemical shifts of viscosinamide A (natural isotope abundance) in acetonitrile-d_3 at 298.0 K.

			^1H δ [ppm]	^{13}C δ [ppm]
HDA				
		CO		174.90
		$CH_2\alpha1$	2.36	44.70
		$CH_2\alpha2$	2.46	44.70
		$CH\beta$	3.97	69.68
		$CH_2\gamma$	1.48	38.10
		$CH_2\delta1$	1.31	26.31
		$CH_2\delta2$	1.45	26.31
		$CH_2\varepsilon$	1.31	30.15
		$CH_2\zeta$	1.29	29.94
		$CH_2\eta$	1.27	32.49
		$CH_2\theta$	1.29	23.30
		$CH_3\iota$	0.88	14.32
		OH	3.59	
L-Leu1				
$^3J_{HNH\alpha}$	5.7 Hz	NH	7.98	
		$CH\alpha$	3.84	53.53
		CO		175.27
		$CH_2\beta1$	1.69	39.09
		$CH_2\beta2$	1.78	39.09
		$CH\gamma$	1.69	25.27
		$CH_3\delta1$	0.89	21.93
		$CH_3\delta2$	0.92	23.20
D-Gln2				
$^3J_{HNH\alpha}$	4.0 Hz	NH	8.72	
		$CH\alpha$	4.07	57.19
		CO		176.43
		$CH_2\beta$	1.98	26.35
		$CH_2\gamma$	2.36	31.91
		$CO\delta$		176.18
		NH2	5.83/6.39	
D-aThr3				
$^3J_{HNH\alpha}$	7.5 Hz	NH	8.10	
		$CH\alpha$	4.04	61.63
		CO		174.32
		$CH\beta$	5.29	70.23
		$CH_3\gamma$	1.28	18.42
D-Val4				
$^3J_{HNH\alpha}$	6.3 Hz	NH	7.75	
		$CH\alpha$	3.39	65.67
		CO		174.13
		$CH\beta$	2.11	29.96
		$CH_3\gamma1$	0.92	19.43
		$CH_3\gamma2$	0.95	20.75
L-Leu5				
$^3J_{HNH\alpha}$	4.3 Hz	NH	8.46	
		$CH\alpha$	3.67	53.80
		CO		171.61
		$CH_2\beta1$	1.73	37.34
		$CH_2\beta2$	1.94	37.34
		$CH\gamma$	1.60	25.53
		$CH_3\delta1$	0.86	21.26
		$CH_3\delta2$	0.92	23.91
D-Ser6				
$^3J_{HNH\alpha}$	8.5 Hz	NH	7.06	
		$CH\alpha$	4.35	56.43
		CO		172.06
		$CH_2\beta1$	3.80	65.01
		$CH_2\beta2$	4.18	65.01
		$OH\gamma$	5.07	
L-Leu7				
$^3J_{HNH\alpha}$	6.5 Hz	NH	7.41	
		$CH\alpha$	4.11	55.03
		CO		N.D.
		$CH_2\beta1$	1.57	41.96
		$CH_2\beta2$	1.93	41.96
		$CH\gamma$	1.92	25.38
		$CH_3\delta1$	$CH_3\delta1$	21.31
		$CH_3\delta2$	0.98	23.20
D-Ser8				
$^3J_{HNH\alpha}$	9.1 Hz	NH	8.00	
		$CH\alpha$	4.45	56.94
		CO		171.71
		$CH_2\beta1$	3.65	63.11
		$CH_2\beta2$	3.85	63.11
		$OH\gamma$	3.93	
L-Ile9				
$^3J_{HNH\alpha}$	10.7 Hz	NH	6.61	
		$CH\alpha$	4.56	57.04
		CO		169.95
		$CH\beta$	1.98	36.73
		$CH_2\gamma1$	0.81	25.10
		$CH_2\gamma2$	0.82	25.10
		$CH_3\gamma1$	0.94	16.12
		$CH_3\delta$	0.86	12.22

Table S3: Chemical shifts of viscosinamide A (natural isotope abundance) in 100 mM DPC-d38 at 298.0 K.

			^{1}H δ [ppm]	^{13}C δ [ppm]				^{1}H δ [ppm]	^{13}C δ [ppm]
HDA					Leu5				
		CO	-	n.d.	$^{3}J_{HNH\alpha}$	n.d.	NH	8.44	-
		CH$_2$α1	2.65	43.59			CHα	3.81	52.85
		CH$_2$α2	2.42	43.57			CO	-	n.d.
		CHβ	4.06	68.31			CH$_2$β1	1.95	36.34
		CH$_2$γ	1.43	36.47			CH$_2$β2	1.66	36.34
		CH$_2$δ1	1.40	25.73			CHγ	1.56	24.69
		CH$_2$δ2	1.28	25.73			CH$_3$δ1	0.88	25.54
		CH$_2$ε	1.22	29.61			CH$_3$δ2	0.84	20.75
		CH$_2$ζ	1.22	29.61	Ser6				
		CH$_2$η	1.20	31.98	$^{3}J_{HNH\alpha}$	n.d.	NH	7.27	-
		CH$_2$θ	1.22	22.60			CHα	4.49	55.51
		CH$_2$ι	0.80	13.89			CO	-	n.d.
		OH	n.d.	-			CH$_2$β1	4.10	62.96
Leu1							CH$_2$β2	3.89	62.96
$^{3}J_{HNH\alpha}$	n.d.	NH	9.37	-			OHγ	n.d.	-
		CHα	3.89	51.82	Leu7				
		CO	-	n.d.	$^{3}J_{HNH\alpha}$	n.d.	NH	7.78	-
		CH$_2$β1	1.75	38.43			CHα	4.53	55.48
		CH$_2$β2	1.57	38.44			CO	-	n.d.
		CHγ	1.71	24.21			CH$_2$β1	1.97	40.98
		CH$_3$δ1	0.86	22.87			CH$_2$β2	1.52	40.98
		CH$_3$δ2	0.86	21.51			CHγ	1.98	24.36
Gln2							CH$_3$δ1	0.94	22.86
$^{3}J_{HNH\alpha}$	n.d.	NH	8.77	-			CH$_3$δ2	0.89	21.01
		CHα	4.01	56.03	Ser8				
		CO	-	n.d.	$^{3}J_{HNH\alpha}$	n.d.	NH	8.20	
		CH$_2$β	2.01	25.52			CHα	4.53	56.03
		CH$_2$γ1	2.40	31.10			CO	-	n.d.
		CH$_2$γ2	2.36	31.10			CH$_2$β1	3.90	61.62
		COδ	-	n.d.			CH$_2$β2	3.74	61.62
		OH	6.80/7.56	-			OHγ	n.d.	-
Thr3					Ile9				
$^{3}J_{HNH\alpha}$	n.d.	NH	7.83	-	$^{3}J_{HNH\alpha}$	n.d.	NH	6.74	-
$^{3}J_{H\alpha H\beta}$		CHα	4.10	60.34			CHα	4.59	56.58
		CO	-	n.d.			CO	-	n.d.
		CHβ	5.32	70.25			CHβ	2.01	35.98
		CH$_3$γ	1.29	17.41			CH$_2$γ1	1.12	15.59
Val4							CH$_2$γ2	0.80	24.18
$^{3}J_{HNH\alpha}$	n.d.	NH	8.10	-			CH$_3$γ1	0.92	24.18
		CHα	3.32	64.76			CH$_3$δ	0.81	11.75
		CO	-	n.d.					
		CHβ	2.18	28.72					
		CH$_3$γ1	0.89	20.20					
		CH$_3$γ2	0.86	18.90					

Table S4: The $^{3}J_{HNHA}$ values obtained in acetonitrile and in DPC solution.

Amino acid	$^{3}J_{HNHA}$ in acetonitrile [Hz]		$^{3}J_{HNHA}$ in DPC solution [Hz]	
	1D 1H	HNHA[a]	HNHA[b]	HNHA[c]
L-Leu1	5.8	6.1	6.3	6.6
D-Gln2	4.2	4.4	3.7	3.9
D-aThr3	7.6	7.5	7.3	7.6
D-Val4	5.9	5.9	5.5	5.7
L-Leu5	6.7	6.7	6.3	6.5
D-Ser6	8.4	8.3	8.1	8.5
L-Leu7	5.9	5.9	5.6	5.8
D-Ser8	9.0	8.8	8.6	9.0
L-Ile9	10.2	10.2	9.7	10.1

[a,b] 'Raw' $^{3}J_{HNHA}$ values directly extracted from the respective HNHA spectrum in acetonitrile and in DPC solution. In DPC solution the values are slightly underestimated due to hindered rotational properties. [c] 'Actual' $^{3}J_{HNHA}$ values that were obtained by the uniform multiplication of the 'raw' values by 1.043. The correction factor was chosen to minimize the discrepancy between the 'raw' $^{3}J_{HNHA}$ values of the residues in the macrocycle (from D-aThr3 up to L-Ile9) obtained in the two different media. (Supplementary Figure S9)
